# Phacoemulsification in adult patients with post-uveitis complicated cataract

**Published:** 2018

**Authors:** Sorin Simion Macarie, Daniela Mariana Macarie

**Affiliations:** * Department of Ophthalmology, “Iuliu Hatieganu” University of Medicine and Pharmacy, Cluj-Napoca, Romania; ** Department of Ophthalmology, Integrated Ambulatory, Clinical Hospital of Infectious Diseases, Cluj-Napoca, Romania

**Keywords:** cataract, phacoemulsification, anterior uveitis, postoperative complications

## Abstract

**Objective:** To evaluate the difficulties, complications and outcome of cataract surgery in patients with complicated cataract after anterior uveitis.

**Methods:** A retrospective study on 37 patients who suffered phacoemulsification surgery for post-uveitis complicated cataract in the period 2009–2014 was performed. 43 eyes underwent surgery.

**Results:** Posterior synechiae were present in 25 eyes. Posterior synechiolysis ensured large pupil in 15 eyes, and, in 14 eyes, the use of iris hooks or pupil expansion ring was necessary for surgery. Posterior capsule rupture was reported in 1 eye. Opacified and thick posterior capsule was noticed in 6 eyes. After surgery, transient rising of intraocular pressure was noticed in 4 eyes and prolonged postoperative inflammation was present in 3 eyes. Posterior capsule opacification was the most frequent issue (9 eyes) and cystoid macular oedema was reported in 2 eyes.

**Conclusions:** Post-uveitis complicated cataract surgery presents specific difficulties and postoperative complications are present in these patients, like prolonged postoperative inflammation and cystoid macular oedema.

## Introduction

Cataract surgery for post-uveitis cataract represents approximately 1,2% of all cataract surgery procedures in adults [**1**]. In most of those patients, eyes present some aspects that can produce either difficulties and complications during surgery, or short or long-term complications. Irregular and small pupil, anterior synechiae, atrophic iris, small anterior chamber, weak capsular bag, or zonulae are some of those aspects. Retinal disorders (macular oedema, retinal scars, vitreoretinal interface pathology) can preexist or can occur after cataract surgery. All those factors can determine poor visual outcome after cataract surgery in these patients. 

## Methods

**Fig. 1:**
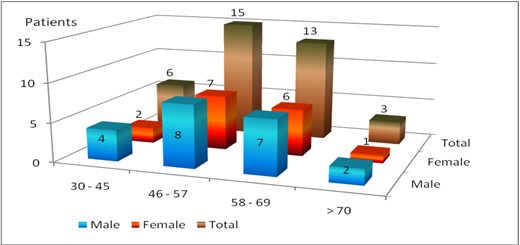
Age and sex distribution of the patients included in our study

A retrospective study on 37 patients, who underwent phacoemulsification (37 eyes) surgery during the period 2009–2014 for complicated cataract after endogen anterior uveitis, was performed. 21 patients were males (56,75%), 16 females (43,25%). Patients’ age and sex distribution is represented in **Fig. 1**. Three patients presented ankylosing spondylitis, one patient presented psoriasis with arthritis, and the others idiopathic anterior uveitis. Phacoemulsification surgery was performed with 2,2 mm incision with Alcon Infiniti and Alcon Constellation systems. All the patients underwent surgery only if they presented a minimum period of 6 months of inactive inflammation. 

## Results 

16 patients (43.24%) presented irregular pupil with 1–3 posterior synechiae, 9 patients (24.32%) presented small pupil, with extended posterior synechiae (24,32%) and 26 patients (70,27%) presented pigment on the anterior capsule of the lens. All the patients presented normal intraocular pressure, 11 patients presented goniosynechiae (29,7%). An accurate retinal examination was not possible in 8 patients and the other patients presented a normal retinal aspect. 

The type and hardness of cataract is presented in **Table 1**. 

**Table 1:** Type of cataract and nucleus hardness of the patients included in our study

Type of cataract / Nucleus hardness	Posterior subcapsular	Nuclear	Cortical and posterior subcapsular	Nuclear and posterior subcapsular 70
+	7	0	5	0
++	2	3	2	3
+++	0	7	0	8

Preoperative dilatation of the pupil was good in 12 patients (32,43%). In 15 patients (37.83%), synechiolysis with cystotome needle allowed us to obtain a good pupil diameter for phacoemulsification. In 10 (27,02%) cases, we had to use iris hooks or perfect pupil rings from the beginning of the surgical procedure: in 4 patients, the pupil became small, despite good initial dilation, and we also had to use iris hooks. In 9 cases (24,32%), we found a thin friable anterior capsule, and 5 patients presented diffuse iris atrophy. Opacified and thick posterior capsule was noticed in 6 eyes (16,21%). 

In 31 patients (83,78%), we had no incidents during surgery. Two patients presented anterior capsule tear after synechiolysis, one patient presented a small anterior chamber bleeding after intraocular lens implantation, and, in two patients, we observed zonulolysis and we had to implant a capsular tensional ring. In one patient, we observed posterior capsule rupture and we had to perform anterior vitrectomy.

After surgery, transient rising of intraocular pressure was noticed in 4 eyes (10,81%) more frequent than in non-uveitis patients, estimated at 0,5% [**2**]. Prolonged postoperative inflammation (more than 14 days) was present in 3 eyes. Posterior capsule opacification was the most frequent issue in the first 6 months after surgery (9 eyes – 24,32%). Cystoid macular oedema was reported in 2 eyes (5,4%) at 4 and 6 months after surgery, more frequent than in senile (non-uveitis) patients, being estimated at 4% [**3**]. One of the patients presented posterior capsule rupture during surgery; the other suffered Nd-YAG laser capsulotomy. 

**Fig. 2:**
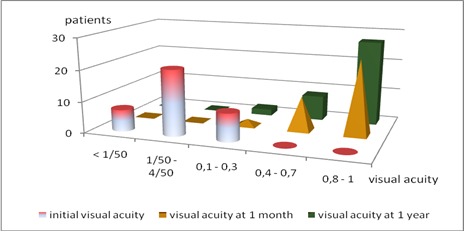
Preoperative and postoperative visual acuity

Preoperative and postoperative visual acuity is presented in **Fig. 2**. 24 patients (64,86%) presented visual acuity better than 0,8 at 1 month after surgery, and 27 (72,9%) at one year. 

## Discussions 

Cataract is a common complication of uveitis [**3**,**4**]. Surgical management of complicated cataract after anterior uveitis needs additional actions, especially for achieving and maintaining a good mydriasis [**4**] or for a good stabilization of the intraocular lens. Posterior capsule opacification is the most frequent issue in these patients [**2**,**5**]. Cystoid macular edema occurs after surgery in the patients who suffered posterior capsule rupture (accidentally or by Nd-YAG laser capsulotomy); some authors state that perioperative systemic therapy is protective against those complications [**6**,**7**]. Prolonged eye inflammation after surgery is relatively rare, especially if heparin-surface-modified intraocular lenses are implanted [**8**]. Postoperative rising of intraocular pressure can occur frequently in these patients [**2**,**9**]. Good preoperative and postoperative control of inflammation plays an important role in achieving favorable visual outcomes [**9**,**10**]. Visual outcome is generally good, but poorer than in non-uveitis eyes [**11**]. 

## Conclusions

Surgery of complicated cataract after anterior uveitis offers good visual outcome in 72,9% of the cases (visual acuity of more than 0.8). Iris hooks or perfect pupil rings are very frequently required in these patients (37,83%). Posterior capsule opacification and cystoid macular edema are more frequent than in senile (non-uveitis) cataract. After surgery, elevated intraocular pressure and prolonged ocular inflammation can occur. 

**Disclosures**

None
